# Supplementary figures and images for: Interelemental osteohistological variation in Massospondylus carinatus and its implications for locomotion
Source: PeerJ. 2022 Sep 23;10:e13918. doi: 10.7717/peerj.13918 (PMC9512004; doi:10.7717/peerj.13918)

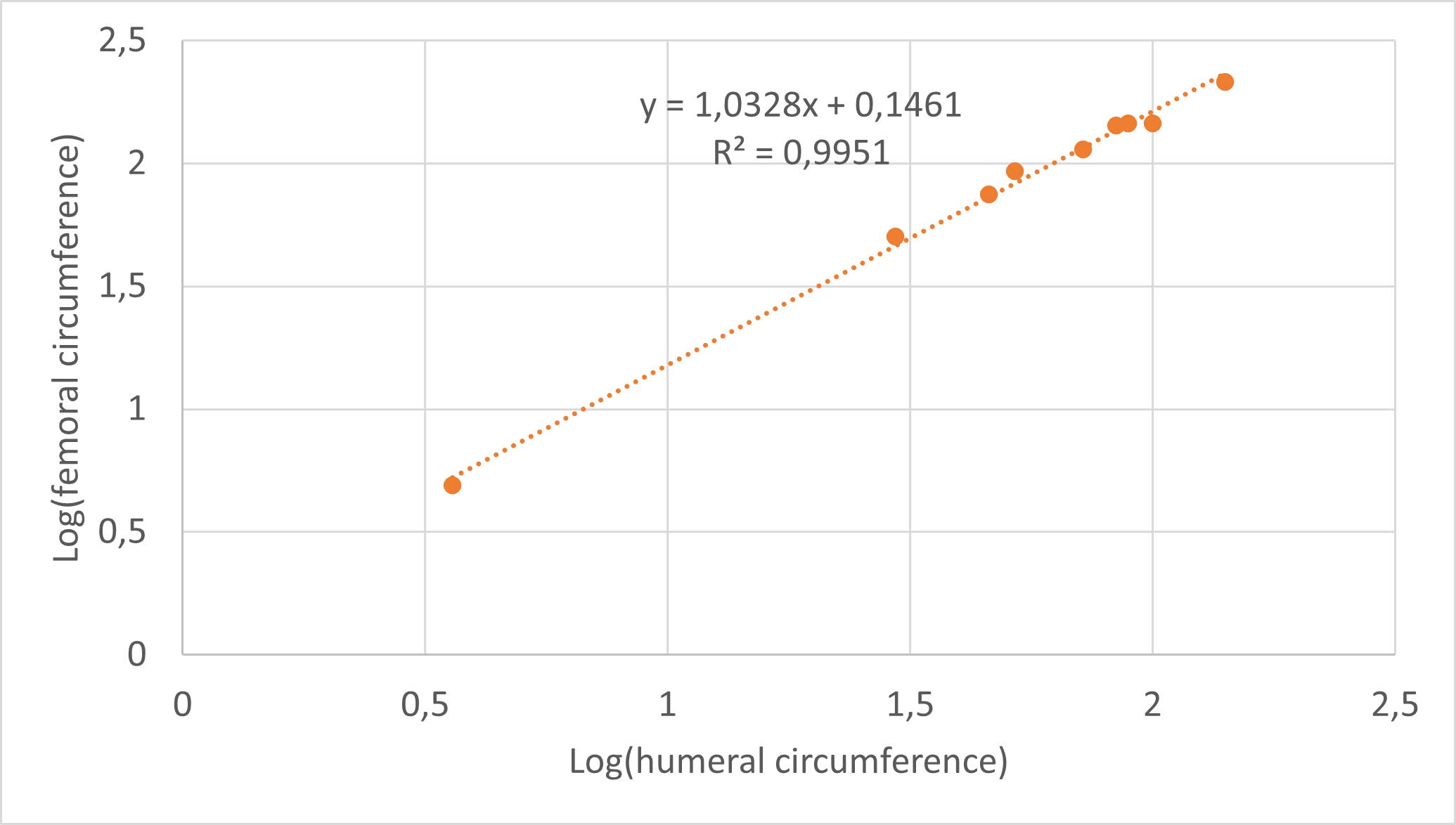

Supplement: Supplemental Information 1 [file peerj-10-13918-s001.png]

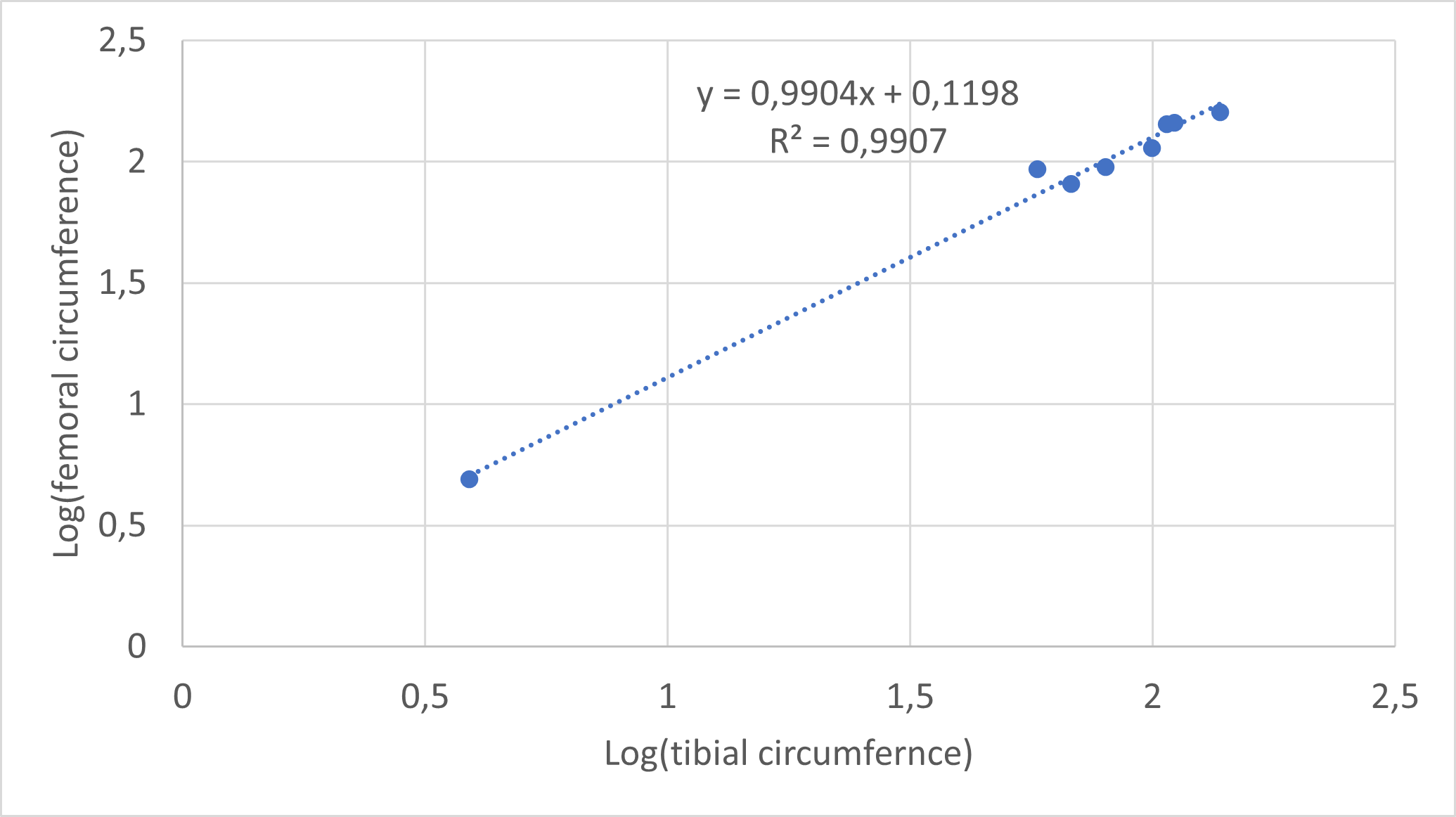

Supplement: Supplemental Information 2 [file peerj-10-13918-s002.png]

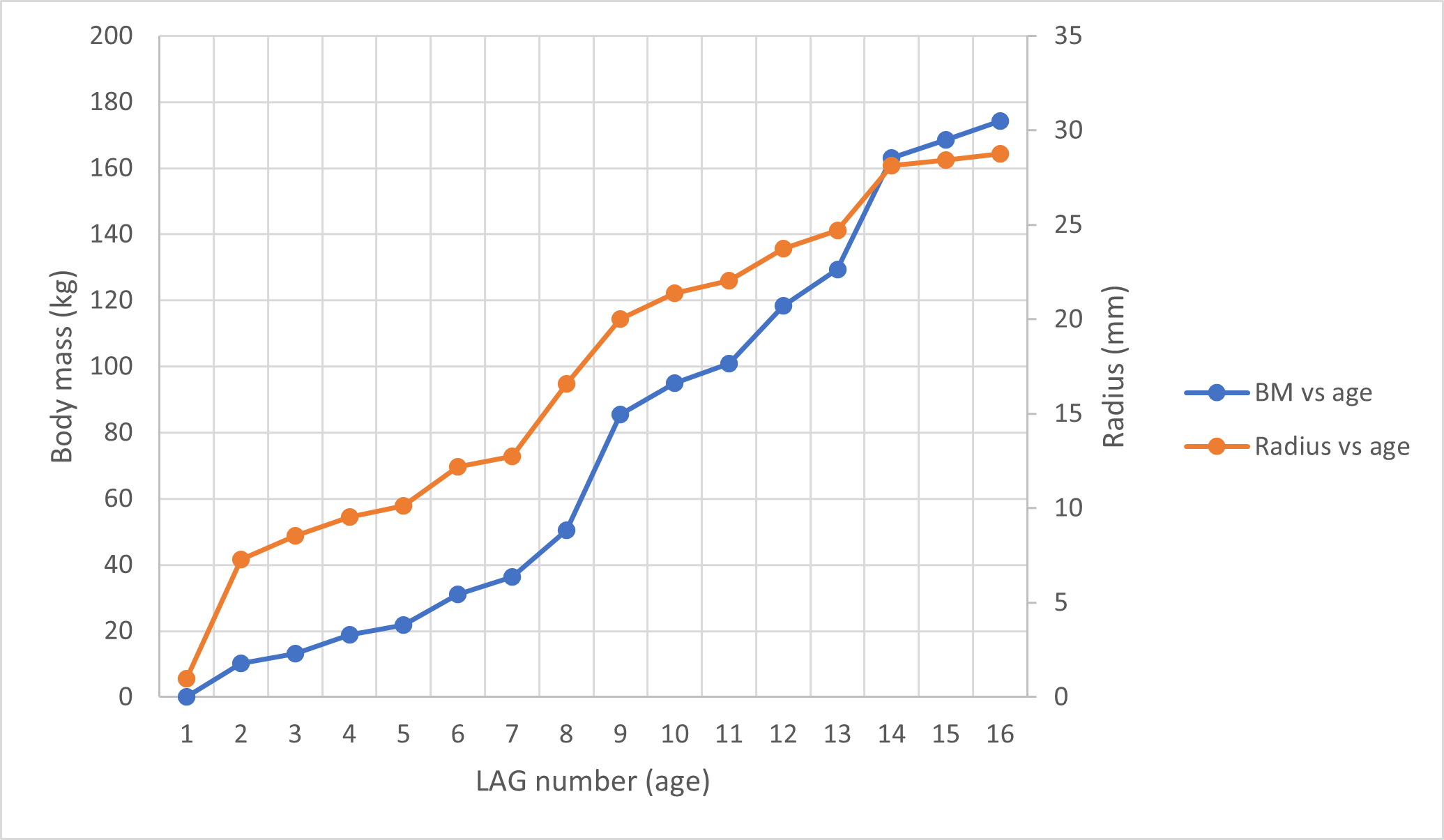

Supplement: Supplemental Information 3 — Abbreviations: BM, body mass. [file peerj-10-13918-s003.png]

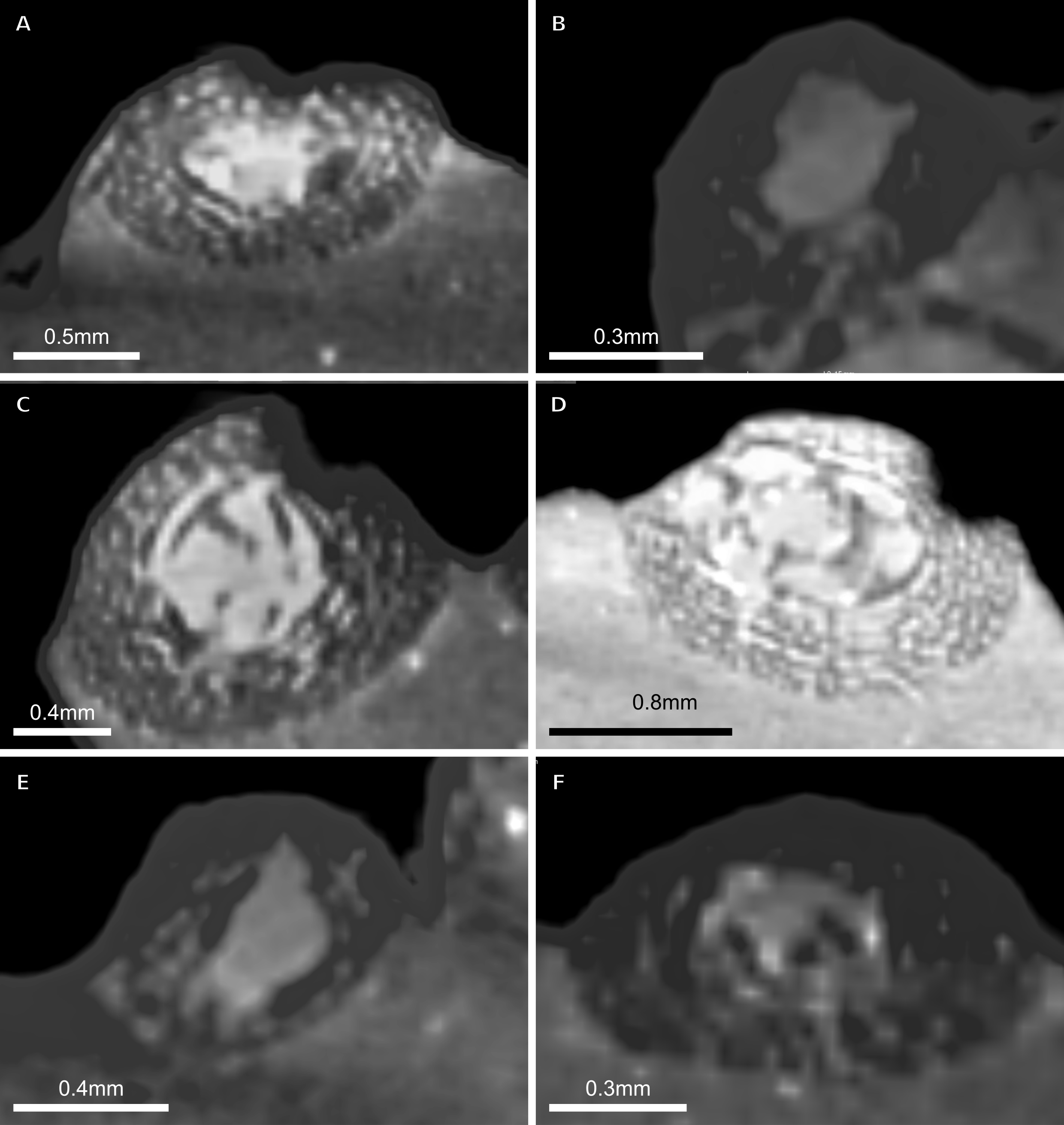

Supplement: Supplemental Information 4 [file peerj-10-13918-s004.png]
