# Supplementary material for: Interelemental osteohistological variation in Massospondylus carinatus and its implications for locomotion
Source: PeerJ. 2022 Sep 23;10:e13918. doi: 10.7717/peerj.13918 (PMC9512004; doi:10.7717/peerj.13918)
Supplement: Supplemental Information 6 [file peerj-10-13918-s006.docx]

| Specimen | LAG number (age) | Circumference (um) | Circumference (mm) | Log10(body mass) | Body mass (g) | Body mass (kg) | LAG radius (mm) |
| --- | --- | --- | --- | --- | --- | --- | --- |
| BP/1/5347 | 1 | 4900 | 4,9 | 1,2178 | 16,51201 | 0,016512 | 0,99 |
| BP/1/5253 | 2 | 50500 | 50,5 | 4,007864 | 10182,74 | 10,18274 | 7,267 |
| BP/1/4266 | 3 | 55382,29 | 55,38229 | 4,118244 | 13129,36 | 13,12936 | 8,52376 |
| BP/1/4266 | 4 | 63081,32 | 63,08132 | 4,273927 | 18790 | 18,79 | 9,538288 |
| BP/1/4266 | 5 | 66528,73 | 66,52873 | 4,337567 | 21755,42 | 21,75542 | 10,13658 |
| BP/1/4266 | 6 | 75797,45 | 75,79745 | 4,493569 | 31157,94 | 31,15794 | 12,18593 |
| BP/1/4266 | 7 | 80215,35 | 80,21535 | 4,561325 | 36418,75 | 36,41875 | 12,74717 |
| BP/1/5241 | 8 | 90277,96 | 90,27796 | 4,702672 | 50428,05 | 50,42805 | 16,59422 |
| BP/1/5241 | 9 | 109344,6 | 109,3446 | 4,931848 | 85476,76 | 85,47676 | 20,01257 |
| BP/1/5241 | 10 | 113598,3 | 113,5983 | 4,977494 | 94949,86 | 94,94986 | 21,39871 |
| BP/1/5241 | 11 | 116142,4 | 116,1424 | 5,003984 | 100921,7 | 100,9217 | 22,04826 |
| BP/1/5241 | 12 | 123092,6 | 123,0926 | 5,073499 | 118440,1 | 118,4401 | 23,74942 |
| BP/1/5241 | 13 | 127057,1 | 127,0571 | 5,111413 | 129244,9 | 129,2449 | 24,69132 |
| BP/1/5241 | 14 | 138243,6 | 138,2436 | 5,212337 | 163056 | 163,056 | 28,11719 |
| BP/1/5241 | 15 | 139950,3 | 139,9503 | 5,227012 | 168659,9 | 168,6599 | 28,43954 |
| BP/1/5241 | 16 | 141622,7 | 141,6227 | 5,24122 | 174269,1 | 174,2691 | 28,75264 |
