# Supplementary material for: Interelemental osteohistological variation in Massospondylus carinatus and its implications for locomotion
Source: PeerJ. 2022 Sep 23;10:e13918. doi: 10.7717/peerj.13918 (PMC9512004; doi:10.7717/peerj.13918)
Supplement: Supplemental Information 8 — Regressions between circumference, cortical thickness, number of LAGs and proportional vascularisation [file peerj-10-13918-s008.docx]

Table S4: Log(cortical thickness) vs Log(estimated and true circumference)

|  | Multiple R^2^ excluding embryo | Adjusted R^2^  excluding embryo | P-value excluding embryo | Multiple R^2^ including embryo | Adjusted R^2^  including embryo | P-value including embryo |
| --- | --- | --- | --- | --- | --- | --- |
| Humerus | 0.3425 | 0.3369 | 2.23e-12 | 0.6884 | 0.6858 | < 2.2*10^-16^ |
| Femur | 0.4433 | 0.4396 | < 2.2e-16 | 0.713 | 0.7111 | < 2.2*10^-16^ |
| Tibia | 0.7197 | 0.7145 | < 2.2e-16 | 0.881 | 0.8789 | < 2.2*10^-16^ |

Table S5: Number of visible CGMs vs circumference

|  | Multiple R^2^ excluding embryo | Adjusted R^2^  excluding embryo | P-value excluding embryo | Multiple R^2^ including embryo | Adjusted R^2^  including embryo | P-value including embryo |
| --- | --- | --- | --- | --- | --- | --- |
| Humerus | 0.6113 | 0.5835 | 0.0003465 | 0.6884 | 0.6858 | < 2.2*10^-16^ |
| Femur | 0.1921 | 0.1445 | 0.06055 | 0.3587 | 0.323 | 0.005269 |
| Tibia | 0.6353 | 0.5745 | 0.01784 | 0.7525 | 0.7172 | 0.002445 |

Table S6: Log(percentage vascularisation) vs Log(estimated or true circumference).

|  | Multiple R^2^ excluding embryo | Adjusted R^2^  excluding embryo | P-value excluding embryo |
| --- | --- | --- | --- |
| Humerus | 0.1772 | 0.0743 | 0.2258 |
| Femur | 0.0266 | -0.07073 | 0.6125 |
| Tibia | 0.1719 | 0.03388 | 0.3071 |

Table S7: Number of humeral CGMs vs number of femoral CGMs and humeral vascularisation vs femoral vascularisation regression coefficients.

|  | Intercept | Slope | Multiple R^2^ | Adjusted R^2^ | P-value |
| --- | --- | --- | --- | --- | --- |
| humeral CGMs vs femoral CGMs | 1.0624 | 0.7861 | 0.7384 | 0.7056 | 0.001443 |
| Humeral vasc vs femoral vsc | 12.9848 | 0.5023 | 0.8299 | 0.7874 | 0.01153 |

Table S8: Regression summary of CGM number vs radius (mm) of overlapping growth series.

|  | Intercept | Slope | Multiple R^2^ | Adjusted R^2^ | P-value |
| --- | --- | --- | --- | --- | --- |
| Humeral LAGs vs radius (mm) | -4.12524 | 0.98263 | 0.9554 | 0.9533 | 1.158*10^-15^ |
| Femoral LAGs vs radius (mm) | -3.6186 | 0.6111 | 0.9619 | 0.96 | 1.157*10^-15^ |
